# Covalent Histone Modification by an Electrophilic Derivative of the Anti-HIV Drug Nevirapine

**DOI:** 10.3390/molecules26051349

**Published:** 2021-03-03

**Authors:** Shrika G. Harjivan, Catarina Charneira, Inês L. Martins, Sofia A. Pereira, Guadalupe Espadas, Eduard Sabidó, Frederick A. Beland, M. Matilde Marques, Alexandra M. M. Antunes

**Affiliations:** 1 Centro de Química Estrutural (CQE), Instituto Superior Técnico, Universidade de Lisboa, 1049-001 Lisbon, Portugal; sharjivan@farm-id.pt (S.G.H.); catarina.charneira@tecnico.ulisboa.pt (C.C.); ines.martins@insa.min-saude.pt (I.L.M.); matilde.marques@tecnico.ulisboa.pt (M.M.M.); 2 Centro de Estudos de Doenças Crónicas (CEDOC), NOVA Medical School, Faculdade de Ciências Médicas, Universidade Nova de Lisboa, 1169-056 Lisbon, Portugal; sofia.pereira@nms.unl.pt; 3 Proteomics Unit, Centre for Genomic Regulation (CRG), Dr. Aiguader 88, 08003 Barcelona, Spain; guadalupe.espadas@crg.eu (G.E.); eduard.sabido@crg.cat (E.S.); 4 Proteomics Unit, Universitat Pompeu Fabra (UPF), Dr. Aiguader 88, 08003 Barcelona, Spain; 5 Division of Biochemical Toxicology, National Center for Toxicological Research, U.S. Food and Drug Administration, Jefferson, AR 72079, USA; frederick.beland@fda.hhs.gov

**Keywords:** covalent histone modification, mass spectrometry, drug-induced adverse reactions, covalent protein adducts, nevirapine

## Abstract

Nevirapine (NVP), a non-nucleoside reverse transcriptase inhibitor widely used in combined antiretroviral therapy and to prevent mother-to-child transmission of the human immunodeficiency virus type 1, is associated with several adverse side effects. Using 12-mesyloxy-nevirapine, a model electrophile of the reactive metabolites derived from the NVP Phase I metabolite, 12-hydroxy-NVP, we demonstrate that the nucleophilic core and *C*-terminal residues of histones are targets for covalent adduct formation. We identified multiple NVP-modification sites at lysine (e.g., H2BK47, H4K32), histidine (e.g., H2BH110, H4H76), and serine (e.g., H2BS33) residues of the four histones using a mass spectrometry-based bottom-up proteomic analysis. In particular, H2BK47, H2BH110, H2AH83, and H4H76 were found to be potential hot spots for NVP incorporation. Notably, a remarkable selectivity to the imidazole ring of histidine was observed, with modification by NVP detected in three out of the 11 histidine residues of histones. This suggests that NVP-modified histidine residues of histones are prospective markers of the drug’s bioactivation and/or toxicity. Importantly, NVP-derived modifications were identified at sites known to determine chromatin structure (e.g., H4H76) or that can undergo multiple types of post-translational modifications (e.g., H2BK47, H4H76). These results open new insights into the molecular mechanisms of drug-induced adverse reactions.

## 1. Introduction

**Scheme 1:**
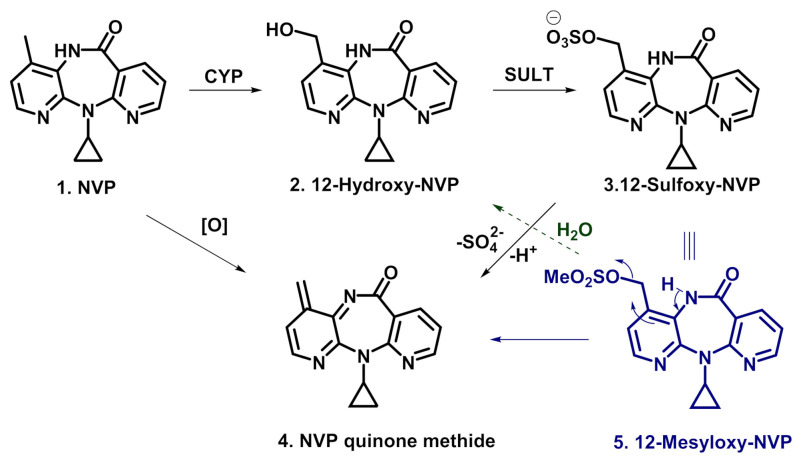
Nevirapine (NVP, **1**), its Phase I metabolite, 12-hydroxy-NVP (**2**), and the bioactivation pathways leading to the Phase II metabolite 12-sulfoxy-NVP (**3**) and to NVP quinone methide (**4**). Additionally shown in blue is the structure of the electrophilic surrogate 12-mesyloxy-NVP (**5**), which not only displays structural similarity with **3** but also yields, at least transiently, the NVP quinone methide. The dashed arrow indicates that (**5**) undergoes ready hydrolysis to (**2**) in aqueous media. CYP, cytochrome P450; SULT, sulfotransferase.

Nevirapine (NVP, **1**, Scheme 1) was the first non-nucleoside reverse transcriptase inhibitor (NNRTI) approved by the U.S. Food and Drug Administration in 1996 for treatment of the human immunodeficiency virus type 1 (HIV-1) [1]. Currently, NVP remains widely used in combined antiretroviral therapeutic regimens and also to reduce mother-to-child transmission of the virus. However, there are multiple reports of adverse effects associated with NVP administration, particularly skin rash and immune-mediated toxicity, which can at times be fatal and often lead to drug cessation [2,3,4,5,6]. Two additional concerns are the observation that rodents under long-term NVP regimens had an increased incidence of hepatocellular adenomas and carcinomas [7], and a reported epidemiological association between chronic NNRTI therapies and the occurrence of non-AIDS-defining cancers in HIV-positive patients [8]. These observations suggest that NNRTIs may be carcinogenic, and emphasize the need to develop efficient analytical tools for accurate estimations of risks/benefits associated with prolonged NVP therapy, particularly in pediatric settings. Although the specific mechanisms of NVP-induced adverse events have yet to be fully elucidated [9,10,11,12], the drug is well documented to undergo bioactivation to reactive metabolites that can modify biomacromolecules [13,14,15]. In line with this evidence, a considerable amount of work has been conducted in the last decade to develop mass spectrometric-based tools for the assessment of covalent NVP-protein and NVP-DNA adducts, which may serve as biomarkers of metabolic activation [13,14,15,16,17,18]. The rationale behind these studies is the recognition that drug bioactivation is frequently at the onset of drug-induced adverse effects, as a consequence of the formation of covalent adducts that may prompt carcinogenic and immune-mediated processes [19].

Following chronic administration of a daily dose of 400 mg, the steady-state maximum plasma concentration of NVP is ca. 7.2 mg/L [12]. The drug undergoes extensive metabolic Phase I transformations affording multiple oxidized metabolites [20]. However, only the bioactivation of the 12-hydroxy-NVP metabolite (**2**, Scheme 1) has thus far been associated with NVP-induced toxic events. The formation of the reactive Phase II metabolite 12-sulfoxy-NVP (**3**, Scheme 1) and/or NVP quinone methide (**4**) [21,22,23] upon bioactivation of 12-hydroxy-NVP has been consistently implicated in the adverse effects elicited by NVP.

The detection of covalent adducts with blood proteins stemming from 12-hydroxy-NVP bioactivation in human patients has been reported by us [14] and by Park’s research group [15]. However, while blood proteins, such as hemoglobin and human serum albumin, are suitable to demonstrate NVP bioactivation in vivo, they are only surrogates for toxicologically relevant proteins; as such, they do not allow direct correlations to be established between the formation of covalent adducts in humans and the occurrence of drug-induced adverse reactions. This limitation prompted us to investigate the presence of NVP-induced covalent modifications in proteins that could combine potential toxicological significance with easy access in vivo by minimally invasive methods.

Histones are nuclear proteins that are determinant in the regulation of chromatin structure and gene expression [24] and can be easily isolated from the buffy-coat and serum fraction of peripheral blood [25,26]. Due to these characteristics, histones are potentially good models for biomonitoring purposes. It is now well recognized that abnormal post-translational modifications (PTMs) of histones play a key role in eliciting anomalies in chromatin structure [27] and function [28], thereby affecting essential functions of many eukaryotic proteins involved in processes such as cellular metabolism, cell cycle, growth, aging, and the etiology of diseases, including cancer [29]. Additionally, recent evidence has increasingly been connecting environmental exposure to toxicants with abnormal epigenetic marks and the development of human diseases [30,31]. Consequently, the search for PTM patterns and their significance is becoming increasingly frequent in toxicological research [32]. However, despite the fact that these lysine-rich proteins are documented targets for reactive species generated endogenously [33,34,35,36,37,38,39], covalent modifications of histones by exogenous toxicants, which may conceivably disturb their normal function, remain relatively unexplored. The first report of covalent in vivo modification of histones by electrophilic metabolites of the food contaminant and rodent carcinogen furan was provided by our group, suggesting that this type of modification by chemical carcinogens may be general in scope [40]. Importantly, the detection of a furan-modified peptide occurred before epigenetic changes could be identified, which suggests that the modification occurred at the initial stages of furan-induced carcinogenesis.

Considering the potential carcinogenicity of NVP [7,8], we conducted a proof-of-concept study, aimed at testing the hypothesis that histones are plausible targets for covalent adduct formation by drug-derived electrophiles. Toward this end, we incubated the human histone octamer and recombinant human histone H4 with 12-mesyloxy-NVP (**5**, Scheme 1), a synthetic surrogate for the reactive electrophilic metabolite **3** and a synthetic precursor of metabolite **4**, both derived from 12-hydroxy-NVP. Multiple sites of histone modification by NVP were identified through the use of an MS-based bottom-up proteomics approach [41].

## 2. Results

### 2.1. Optimization of Digestion Conditions

A critical issue in histone MS analysis is the length of the peptides afforded upon digestion [42,43]. Therefore, our first step was to optimize the digestion conditions. Classic tryptic and Lys-C digestion of histones usually yield very small peptides that are unsuitable for efficient MS sequence analysis, due to the high arginine and lysine content of histone proteins, particularly at the *N*-terminal tails. However, since lysine residues were expected to be targets for NVP modification and the presence of modified lysines could conceivably protect these residues from tryptic cleavage, we decided to assess variations of classic digestion conditions. Optimization of the trypsin digestion conditions was performed with the NVP-modified H4 histone sample using a 2 h trypsin digestion with 1:10, 1:100 and 1:1000 (*w/w*) trypsin:histone ratios. Based on the results obtained (cf. Supplementary Materials Table S1), we chose to use two distinct digestion conditions for subsequent comparative analyses between replicates and between free histones and the histone octamer (cf. Supplementary Materials Tables S2–S4 and Figures S1 and S2): (i) A 15 min trypsin digestion with a 1:100 trypsin:histone ratio, to maximize the information about peptides in the *N*-terminal tails; (ii) a 2 h trypsin digestion with a 1:10 trypsin:histone ratio, to maximize the identification of NVP-derived modifications occurring in the histone cores.

Although the analysis of samples obtained upon a 15 min digestion at a 1:100 trypsin:histone ratio consistently allowed the identification of peptides from the histone tails, NVP modifications were not detected in these regions, despite the use of a large excess of electrophile. For instance, the tail peptide ^10^GLGKGGAKR^18^ of histone H4 was detected, but the corresponding NVP-modified peptide could not be identified, despite the presence of two potential target residues, K13 and K17 (cf. Supplementary Materials, Figure S1). In contrast, the 2 h trypsin digestion with a 1:10 enzyme/histone ratio, which we previously used successfully for the identification of covalent furan- and glycidamide-derived histone modifications, in vivo and in vitro in cells, respectively [40,44], allowed the identification of NVP-derived modifications in all histone samples analyzed.

### 2.2. Identification of NVP-Derived Binding Sites in Histones

**Figure 1:**
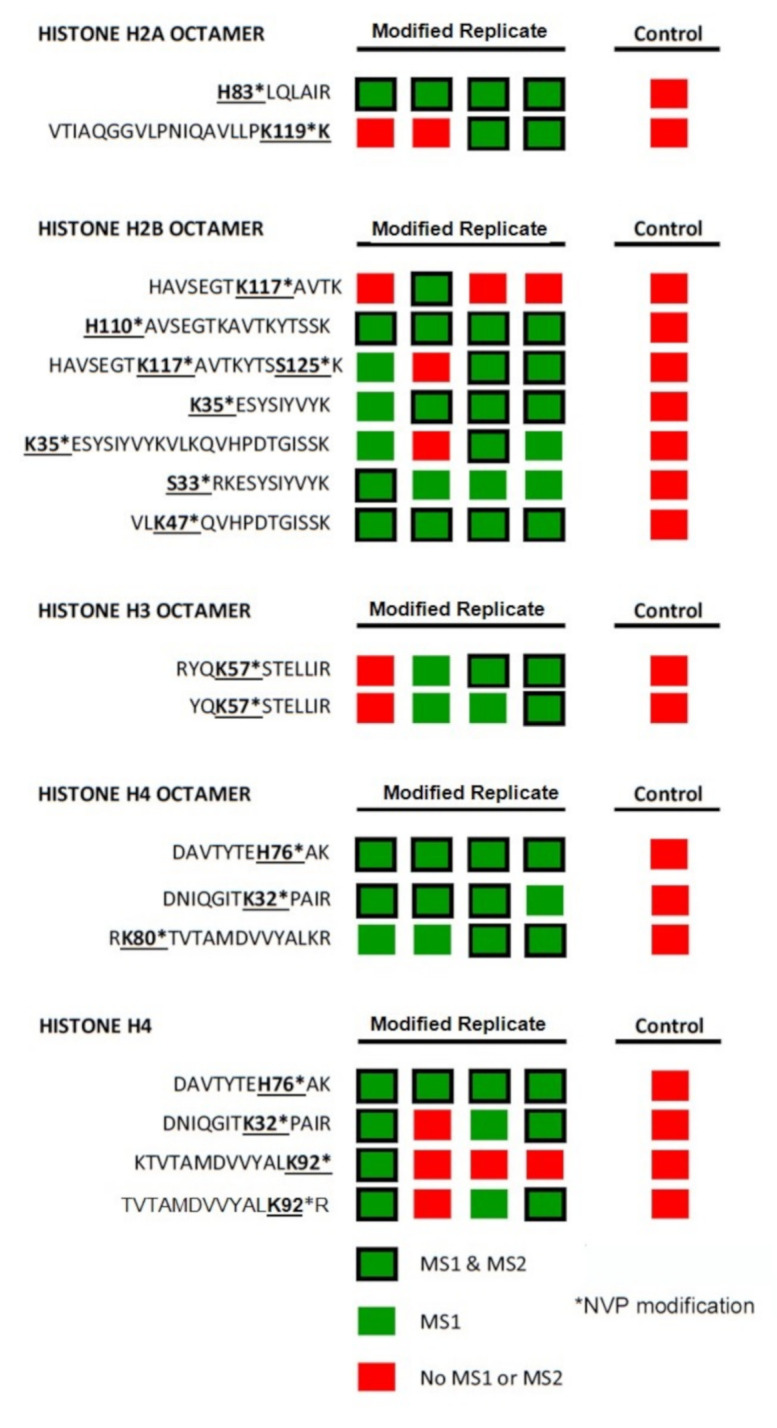
Sequences of nevirapine-modified peptides identified in histones H2A, H2B, H3 and H4, either as purified proteins (H4) or in the octamer structure. Modified peptides observed in each of four replicates are highlighted in green, and a bold border indicates that MS2 information is also present. Modified peptides for which no MS2 evidence was present were identified by ionogram alignment and extraction of the MS1 ion current. Digestion conditions: Two hour trypsin digestion with a 1:10 trypsin:histone ratio.

**Figure 2:**
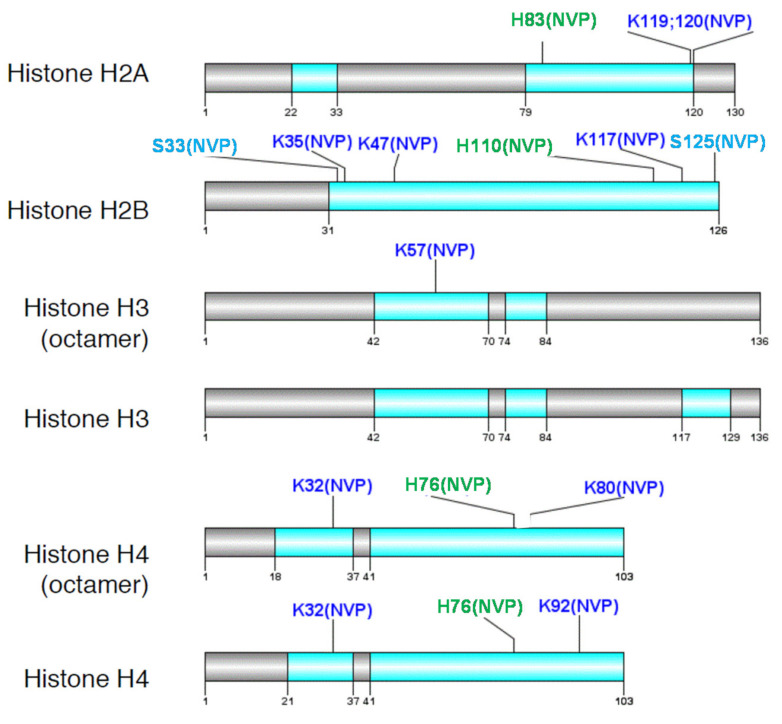
Overview of the H2A, H2B, H3, and H4 sequences showing the nevirapine-modified residues identified in this study. The amino acid number notation takes into account the first methionine, which is removed after protein synthesis. Regions of the sequence covered by MS analysis are represented in light blue; a gray shading represents non-covered regions.

NVP-derived modifications were detected in lysine, serine, and histidine residues of the histone cores and *C*-terminal ends. As expected, they were completely absent from the control samples (Figure 1 and Figure 2). A comparative study of the NVP-derived modifications detected in four replicate incubations, along with the comparison of modifications occurring in the free histone H4 and the histone octamer, was performed using quantitative MS1/MS2 proteomic analysis, upon Skyline MS1 filtering (cf. Supplementary Materials, Figures S1 and S2) [45].

The presence of covalently-bound NVP moieties increased the lipophilicity of the modified peptides, which resulted in increased retention times when compared with their non-modified counterparts. For instance, the peptide ^110^HAVSEGTKAVTKYTSSK^126^ of histone H2B, which was detected in all replicates of the octamer, eluted at 17.03 min, while the corresponding peptide modified with NVP at the H110 residue, ^110^H*AVSEGTKAVTKYTSSK^126^, eluted at 22.12 min (cf. Materials and Methods for the elution conditions).

**Figure 3:**
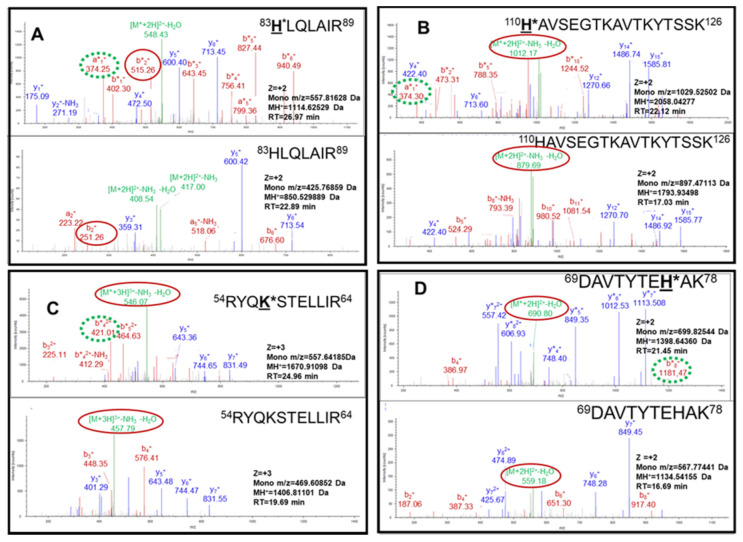
Mass spectra corresponding to representative unmodified peptides and their nevirapine (NVP)-modified counterparts from histones H2A (**A**), H2B (**B**), H3 (**C**), and H4 (**D**). Asterisks (*) indicate NVP-modified residues. Selected red outlines represent fragment ions from NVP-modified peptides and their unmodified counterparts that clearly indicate the NVP mass increment. Dashed green outlines correspond to diagnostic fragment ions that attest the modification site.

The assignment of the amino acid to which NVP was linked was determined from the MS/MS spectrum of each peptide (Figure 3). For example, for the peptide ^110^HAVSEGTKAVTKYTSSK^126^ in histone H2B, the position of the NVP modification was confirmed based on the tandem profile of the dicharged ion at *m/z* 1029.5250 (Figure 3B), which displayed y ions up to y^15^ that were identical to the ones of the non-modified peptide (dicharged ion at *m*/*z* 897.4711). The 264.1005 Da mass increment, characteristic of NVP incorporation, could be observed in the a^1+^ and b^2+^ ions of the NVP-modified peptide, which confirmed H110 as the NVP-binding site. Similar analyses for the peptide ^83^HLQLAIR^89^ in histone 2A indicated NVP modification at H83 (Figure 3A), while peptide ^54^RYQKSTELLIR^64^ in histone 3 was modified at K57 (Figure 3C), and peptide ^69^DAVTYTEHAK^78^ in histone 4 was modified at H76 (Figure 3D).

## 3. Discussion

12-Mesyloxy-NVP (**5**, Figure 1) is a synthetic surrogate of 12-sulfoxy-NVP (**3**) and a synthetic precursor of NVP quinone methide (**4**), which are both reactive metabolites derived from the Phase I NVP metabolite 12-hydroxy-NVP (**2**). In fact, considering their structural similarity, comparable regioselectivities for modification of biomacromolecules can be expected from the potential skin toxicant 12-sulfoxy-NVP [21] and 12-mesyloxy-NVP. Additionally, **5** carries an excellent leaving group at the benzyl-like position and, similarly to quinone methide formation from halide and ester derivatives of *para*- and *ortho*-substituted benzyl alcohols [46], it is anticipated to generate NVP quinone methide (**4**, Scheme 1), the reactive NVP metabolite thought to be implicated in NVP-induced hepatotoxicity [22]. Coherently, this synthetic surrogate has already been very effective as a tool to help characterize NVP adducts formed in vivo. Indeed, the *N*-acetyl-cysteine adducts through NVP-C12 found in rats and humans [13], the adducts at the *N*-terminal valine of hemoglobin identified in humans [14], and the bovine serum albumin adducts obtained upon incubation of NVP with rat hepatocyte spheroids [23] were identical to the fully characterized standards obtained upon in vitro modification of these bionucleophiles with the synthetic model electrophile **5** [17,18,23]. To investigate whether histones can be protein targets of electrophiles derived from 12-hydroxy-NVP, and with the ultimate goal of providing a comprehensive assessment of all possible sites of adduction, we incubated an excess of 12-mesyloxy-NVP with commercially available recombinant full-length human histone H4 and the full-length human histone octamer. Taking into consideration the fact that 12-mesyloxy-NVP (**5**) and its quinone-methide derivative (**4**) are readily hydrolyzed in aqueous media, yielding 12-hydroxy-NVP (**2**), an excess of **5** in the incubations was required to maintain a sufficient amount of electrophile in solution and mimic, to some extent, the cumulative exposure of proteins to NVP-derived electrophiles that will occur in vivo. The global assessment of adduction sites performed in this work represents a key step for subsequent studies involving the search for NVP-derived histone modifications in vivo, in animal models and human samples. We used both full-length histone H4 and the histone octamer to determine if the conformation within the octamer would alter the regioselectivity of the modification sites. Incubations were conducted by an adaptation of the methodology we used previously to modify human serum albumin and hemoglobin with 12-mesyloxy-NVP [18]. To identify the residues containing NVP-derived covalent modifications, we used a proteomics bottom-up approach where, upon appropriate digestion, the resulting crude peptides were analyzed by liquid chromatography-electrospray ionization tandem mass spectrometry (LC-ESI-MS/MS). Specifically, the NVP-modified peptides were expected to contain a mass increment of 264.1005 Da, characteristic of NVP incorporation, when compared with the corresponding peptides obtained from control histones.

**Figure 4:**
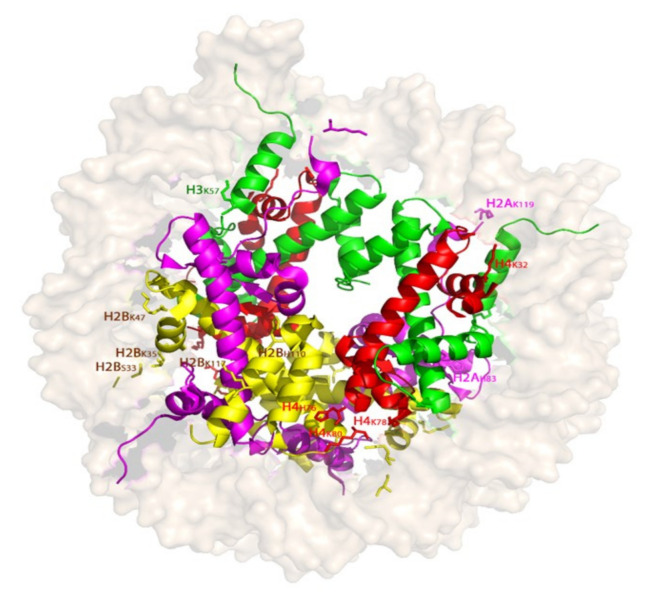
Depiction of the nucleosome, showing the surface exposure of the amino acids found to form adducts with nevirapine. The structure was obtained from PDB 1AOI.

With few exceptions, the NVP modification sites identified in histone H4 of the octamer were the same as those identified in the samples from the reactions with free histone H4 (Figure 2). This suggests that the conformation adopted within the octamer does not influence, to a great extent, the access of electrophiles to the more nucleophilic sites of these proteins. A larger number of adduction sites were identified in histone H2B. This preference was also observed with 4-oxo-2-nonenal-[35], as well as furan- and glycidamide-induced [40,44] covalent modifications, thereby suggesting that histone 2B is a major target for non-enzymatic adduction. The majority of the NVP modifications (Figure 2) occurred in lysine residues, which is consistent with the fact that lysines are the most abundant nucleophilic amino acids of histones. Moreover, all the NVP-modified residues are located on the exposed surface of the proteins (Figure 4) and, remarkably, all lysine residues found to be modified with NVP (Figure 3) have been reported as formylation targets [47,48].

In addition to intranuclear functions, histones also act as damage-associated molecules that activate the immune system, thereby causing further cytotoxicity [49]. Interestingly, high serum levels of anti-H2B antibodies have been identified in HIV patients, accompanied by high CD4^+^ cell titers [50]. It may; therefore, be toxicologically significant that histone H2B was the histone with the most NVP-modification sites identified in this study. The covalent modification of histones by NVP may conceivably result in a potent antigen, similarly to what was observed with histones covalently modified with methylglyoxal [51]. Taking into consideration the strong association between NVP toxicity and higher CD4^+^ cell counts [52], along with the immune nature of these adverse outcomes, the covalent modification of extranuclear histones by NVP-derived electrophiles may be one possible explanation for the onset of adverse events elicited by this anti-HIV drug during the first weeks of treatment.

In this regard, it should be noted that the data-dependent analysis performed in this study is biased toward the most abundant peptides [53]. Therefore, whereas we have used a large excess of electrophile, the most abundant peptides will be those that consistently present MS2 information in the four replicates analyzed. Notably, histidines and H2BK47 were the only modification sites consistently identified with MS2 information in the four replicates. This suggests that, from all potential NVP-binding sites characterized in the present study, H2BK47, H2BH110, H2AH83 and H4H76 are the ones expected to be more prone to NVP incorporation. Also noteworthy is the fact that, although histidines are not very abundant residues in histone proteins, 27% of the histidine residues were consistently identified as sites of NVP modification in the four replicates analyzed (Figure 2). Indeed, while histone H4 contains only two histidines, H76 was consistently identified as an NVP modification site, both in the free histone and the octamer. Similarly, histone H2A contains only four histidine residues, and NVP-modified H83 of histone H2A was consistently identified in the octamer. Likewise, among the three histidine residues of histone H2B, H110 was identified as an NVP-modified residue in the four replicates. Notably, H110 of histone H2B has been reported to form a Michael adduct with 4-oxo-2-nonenal [35]. Our results suggest a remarkable affinity of the histidine imidazole ring toward NVP-derived electrophiles, which is in line with the report by Meng et al. [15] that histidines are the main sites for in vitro modification of human serum albumin (HSA) by the NVP metabolite 12-sulfoxy-NVP, an observation further supported by their identification of an NVP-modified histidine residue in HSA of HIV-positive patients treated with NVP.

NVP-induced modifications were identified in positions (e.g., H2BK47, H2AK119/K120, H3K57, H2BK35, H2BK117, H4H76, H2BS33, and H4K80) that can undergo multiple types of PTMs, primarily methylation and acetylation, but also phosphorylation [48,54,55,56,57]. Should this occur in vivo, and contrary to enzymatically-promoted PTMs, NVP modification of these positions is anticipated to be refractory to repair by “eraser” enzymes [38]. As a consequence, the presence of persistent covalent adducts may prevent key epigenetic modifications, thereby altering the histone code, affecting downstream gene expression, and causing irreversible perturbations of cellular homeostasis. In this regard, it is noteworthy that the occurrence of altered histone methylation patterns in rats administered NVP was reported in a preliminary study [58]. If confirmed in vivo, these types of alterations may be general in scope, thereby providing a plausible molecular link to multiple reports of aberrant epigenetic status associated with exposure of cells and animal models to chemical agents. Additionally, the NVP modification of histone position H4K80, which is involved in histone-DNA interactions [59,60], or of histone position H4H76, involved in the stabilization of the histone octamer [61], might influence the DNA accessibility along with the octamer and chromatin structure. Should this pattern be reproduced in vivo, marked interferences in transcription, DNA damage repair, chromatin structure, chromatin assembly, and heterochromatic gene silencing may arise upon NVP-induced histone modification.

## 4. Materials and Methods

### 4.1. Chemicals

Recombinant full length human histones H3 and H4 (Cat # SRP0177 and SRP0178, respectively), and recombinant full length human histone octamer (Cat # SRP0408), all expressed in *Escherichia coli*, were purchased from Sigma-Aldrich Química, S.A. (Madrid, Spain). All other commercially available reagents and enzymes were acquired from the same supplier, unless specified otherwise, and used as received. 12-Mesyloxy-NVP was prepared as described in Antunes et al. [16].

### 4.2. Modification of Histones H3 and H4 and the Histone Octamer

A solution of 12-mesyloxy-NVP (20 μg) in tetrahydrofuran (1 μL) was added to an aqueous solution (7–10 μL) of each histone (10 μg). The resulting reaction mixture was incubated for 72 h at 37 °C. Non-bonded materials were subsequently removed using Amicon^®^ Ultra Centrifugal Filters (10 kDa, 0.5 mL, 2000 g, 2 min), to prevent NVP-derived post-digestion modification, and the proteins were lyophilized and stored at −80 °C until further use. The modification reactions were conducted in quadruplicate. Control samples were prepared in the same way, except that 12-mesyloxy-NVP was absent from the incubations.

### 4.3. Sample Preparation

The NVP-modified proteins were dissolved in 10 µL of water and, following preliminary experiments to optimize the conditions, aliquots (3.5 µL) were digested with sequencing grade modified trypsin (Promega, Madison, WI, USA; Cat # V5113) in a 100 mM ammonium bicarbonate buffer (pH 8) for either 15 min (with a 1:100 *w*/*w* trypsin:histone ratio) or 2 h (with a 1:10 *w/w* trypsin:histone ratio). The digestions were quenched by the addition of trifluoroacetic acid (10% *v/v*).

After digestion, the tryptic peptides were analyzed using an LTQ-Orbitrap XL mass spectrometer (Thermo Fisher Scientific, Inc., Waltham, MA, USA) coupled to an Agilent 1200 Series nanoLC system (Agilent Technologies, Inc., Santa Clara, CA, USA). The peptide mixtures were loaded onto a Zorbax 300SB-C18 pre-column (Agilent Technologies, Cat # 5065-9913) and were separated by reversed-phase chromatography using a 12 cm column with an inner diameter of 75 μm, packed with 5 μm C18 particles (Nikkyo Technos Co., Ltd., Tokyo, Japan). The chromatography was conducted at a flow rate of 300 nL/min, starting with 97% of 0.1% formic acid in water (Buffer A) and 3% of 0.1% formic acid in acetonitrile (Buffer B). A linear gradient was applied to reach 90% Buffer A/10% Buffer B in 1 min, and then 55% Buffer A/45% Buffer B in 30 min. After each analysis, the pre-column and column were washed for 10 min with 10% Buffer A/90% Buffer B.

### 4.4. Mass Spectrometry

The mass spectrometer was operated in the positive ionization mode with the nano-spray voltage set at 2.5 kV and the source temperature at 200 °C. Ultramark 1621 for the FT mass analyzer was used for external calibration prior to the analyses. An internal calibration was also performed using the background polysiloxane ion signal at *m*/*z* 445.1200. The instrument was operated in data dependent acquisition (DDA) mode and full MS scans, with 1 microscan at a resolution of 60,000, were used over an *m/z* range of 200–2000, with detection in the Orbitrap. The auto gain control (AGC) was set to 10,000, the dynamic exclusion to 60 s, and the charge state filter, to disqualify singly charged peptides, was activated. In each cycle of DDA analysis, and following each survey scan, the 10 most intense ions with multiply charged ions above a threshold ion count of 5000 were selected for fragmentation at a normalized collision energy of 35%. Fragment ion spectra produced via collision-induced dissociation (CID) were acquired in the Ion Trap, the AGC was set to 50,000, and the isolation window to *m*/*z* 2.0; an activation time of 0.1 ms and a maximum injection time of 100 ms were used. All data were acquired with Xcalibur software v2.2 (Thermo Fisher Scientific, Inc., Waltham, MA, USA).

### 4.5. Data Analysis

The Proteome Discoverer software suite (v1.4.0.288, Thermo Fisher Scientific, Inc.) and the Mascot search engine (v2.3, Matrix Science Ltd., London, UK [62]) were used for peptide identification. The data were searched against an in-house generated database containing all Swissprot human database and most common contaminants (>20,000 entries). A precursor ion mass tolerance of 7 ppm at the MS1 level was used and up to six missed-cleavages were allowed. The fragment ion mass tolerance was set to 0.5 Da. Oxidation of methionine, protein acetylation at the *N*-terminal, and NVP (mass increment of 264.1005 Da at lysines, cysteines, serines, and histidines) were set as variable modifications. Only peptides identified with a Mascot Ion Score above 20 were considered. All spectra corresponding to NVP-modified peptides were manually checked.

## 5. Conclusions

As a proof-of-concept study, we identified multiple modification targets in histones exposed to the model electrophile 12-mesyloxy-NVP. Using an MS-based bottom-up proteomics approach, we demonstrated that H2BK47, H2BH110, H2AH83, and H4H76 are the positions more prone to NVP incorporation. Of note, whereas histones have a low content of histidine residues, a significant proportion of these residues (27%) were consistently identified as NVP-binding sites. This suggests a remarkable affinity of the histidine imidazole ring toward NVP-derived electrophiles, which may provide a toxicologically relevant biomarker of NVP bioactivation.

If confirmed in vivo, the occurrence of NVP modifications in sites that are also targets for PTMs (e.g., H2BK47 and H4H76) or key for nucleosome stability (e.g., H4H76) may be translated into an altered histone code, along with marked interferences in chromatin assembly and gene expression, opening new insights into the mechanisms of drug-induced adverse effects. Additionally, the use of easily accessible histone proteins, complementary to the ones currently most used for biomonitoring purposes, could be of considerable interest in view of the potential toxicological significance of histone modification by exogenous electrophiles.

## Data Availability

The mass spectrometry proteomics data have been deposited to the ProteomeXchange Consortium via the PRIDE partner repository with the dataset identifier PXD023751.

## Supplementary Materials

The Supplementary Materials are available online. Table S1, presenting the results obtained upon a 2 h trypsin digestion of NVP-modified histone H4 using 1:10, 1:100, and 1:1000 (*w/w*) enzyme:protein ratios; Supplementary Information Tables S2–S4, presenting the results obtained upon 2 h trypsin digestions (with a 1:10 ratio) and 15 min trypsin digestions (with a 1:100 ratio) of the NVP-modified octamer, H4 and H3, respectively; Supplementary Information Figures S1 and S2, showing comparisons of NVP-modified peptides and *N*-terminal peptide identification by Proteome Discoverer and Skyline MS1 filtering in the controls, free histone H4 and histone H4 of the NVP-modified octamer, as well as the Skyline results for each NVP-modified peptide.

## Sample Availability

Samples of the compounds **2** and **5** are available from the authors.
