# Supplementary material for: Covalent Histone Modification by an Electrophilic Derivative of the Anti-HIV Drug Nevirapine
Source: Molecules. 2021 Mar 3;26(5):1349. doi: 10.3390/molecules26051349 (PMC7961589; doi:10.3390/molecules26051349)
Supplement: Supplementary file 1 [file molecules-26-01349-s001.zip › Supplementary Information_molecules-1078589/Supplementary Information Figures S1 and S2.pptm]

## Slide 1
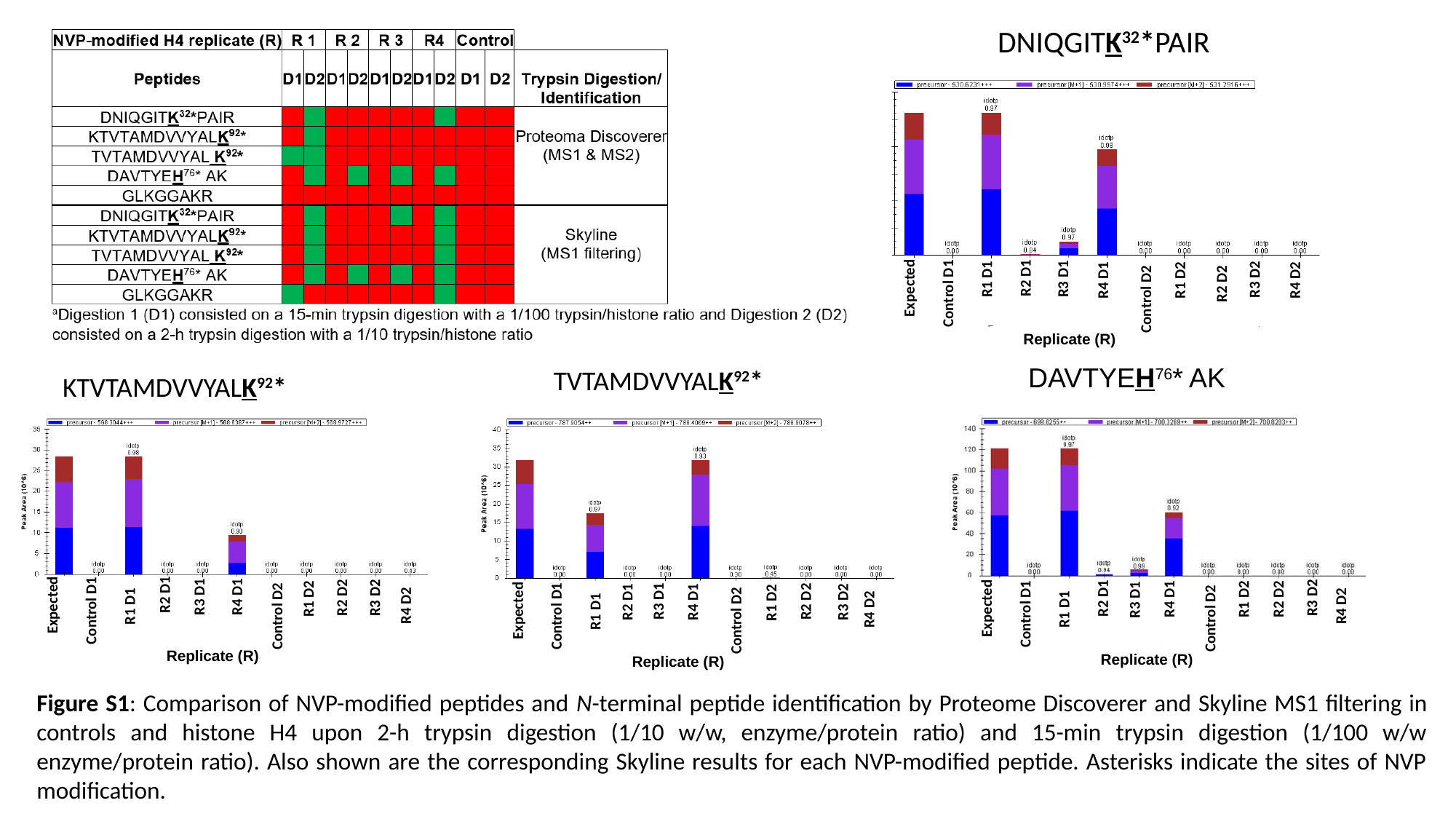

DNIQGITK32*PAIR
Expected
 Control D1
 R2 D1
 R3 D1
 R1 D1
 Control D2
 R3 D2
 R4 D1
 R1 D2
 R4 D2
 R2 D2
Replicate (R)
DAVTYEH76* AK
TVTAMDVVYALK92*
KTVTAMDVVYALK92*
 R2 D1
Expected
 Control D1
 R4 D2
 R4 D2
 R1 D1
 R3 D1
 R4 D1
 Control D2
Expected
 Control D1
 R2 D1
 R2 D2
 R3 D2
 R3 D2
 Control D2
 R4 D1
 R1 D2
 R4 D2
 R1 D1
 R3 D1
 R2 D2
Expected
 R1 D2
Expected
 Control D1
 R3 D1
 Control D2
 R2 D1
 R4 D1
 R1 D1
 R3 D2
 R2 D2
 R1 D2
Replicate (R)
Replicate (R)
Replicate (R)
Figure S1: Comparison of NVP-modified peptides and N-terminal peptide identification by Proteome Discoverer and Skyline MS1 filtering in controls and histone H4 upon 2-h trypsin digestion (1/10 w/w, enzyme/protein ratio) and 15-min trypsin digestion (1/100 w/w enzyme/protein ratio). Also shown are the corresponding Skyline results for each NVP-modified peptide. Asterisks indicate the sites of NVP modification.

## Slide 2
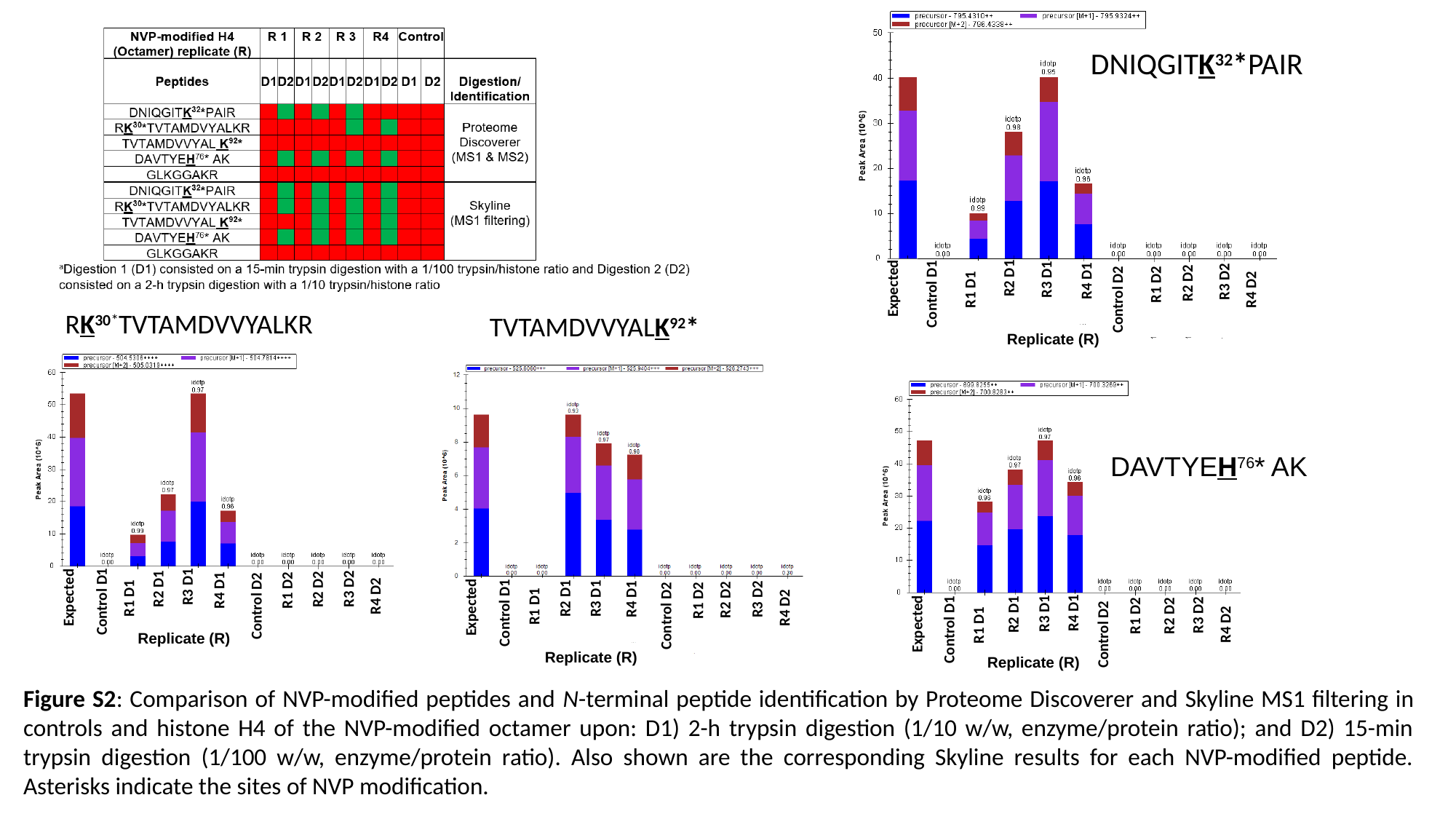

DNIQGITK32*PAIR
Expected
 Control D1
 R2 D1
 R3 D1
 R4 D2
 R1 D1
 Control D2
 R4 D1
 R3 D2
 R2 D2
 R1 D2
RK30*TVTAMDVVYALKR
TVTAMDVVYALK92*
Replicate (R)
DAVTYEH76* AK
 Control D1
 R4 D2
 Control D2
Expected
 R3 D1
 R1 D1
 R2 D1
 R3 D2
 R2 D2
 R4 D1
 R1 D2
 R1 D1
Expected
 Control D1
 R4 D2
 Control D2
 R2 D1
 R3 D1
 R4 D1
 R3 D2
 R2 D2
 R1 D2
 R4 D1
Expected
 R3 D1
 Control D1
 R4 D2
 R2 D1
 R1 D1
 R3 D2
 Control D2
 R1 D2
 R2 D2
Replicate (R)
Replicate (R)
Replicate (R)
Figure S2: Comparison of NVP-modified peptides and N-terminal peptide identification by Proteome Discoverer and Skyline MS1 filtering in controls and histone H4 of the NVP-modified octamer upon: D1) 2-h trypsin digestion (1/10 w/w, enzyme/protein ratio); and D2) 15-min trypsin digestion (1/100 w/w, enzyme/protein ratio). Also shown are the corresponding Skyline results for each NVP-modified peptide. Asterisks indicate the sites of NVP modification.
